# Establishment of patient-derived non-small cell lung cancer xenograft models with genetic aberrations within *EGFR*, *KRAS* and *FGFR1*: useful tools for preclinical studies of targeted therapies

**DOI:** 10.1186/1479-5876-11-168

**Published:** 2013-07-10

**Authors:** Xu-chao Zhang, Jingchuan Zhang, Ming Li, Xiao-sui Huang, Xue-ning Yang, Wen-zhao Zhong, Liang Xie, Lin Zhang, Minhua Zhou, Paul Gavine, Xinying Su, Li Zheng, Guanshan Zhu, Ping Zhan, Qunsheng Ji, Yi-long Wu

**Affiliations:** 1 Medical Research Center of Guangdong General Hospital & Guangdong Academy of Medical Sciences, Guangdong Lung Cancer Institute Guangzhou 510080, PR China; 2 Innovation Center China, AstraZeneca Global R&D, Zhangjiang Hi-Tech Park Shanghai 201203, PR China

**Keywords:** NSCLC, Patient-derived NSCLC xenograft, *EGFR/KRAS* mutations, *FGFR*1 amplification

## Abstract

**Background:**

Patient-derived tumor xenograft models have been established and increasingly used for preclinical studies of targeted therapies in recent years. However, patient-derived non-small cell lung cancer (NSCLC) xenograft mouse models are relatively few in number and are limited in their degree of genetic characterization and validation. In this study, we aimed to establish a variety of patient-derived NSCLC models and characterize these for common genetic aberrations to provide more informative models for preclinical drug efficacy testing.

**Methods:**

NSCLC tissues from thirty-one patients were collected and implanted into immunodeficient mice. Established xenograft models were characterized for common genetic aberrations, including detection of gene mutations within *EGFR* and *KRAS,* and genetic amplification of *FGFR1* and *cMET*. Finally, gefitinib anti-tumor efficacy was tested in these patient-derived NSCLC xenograft models.

**Results:**

Ten passable patient-derived NSCLC xenograft models were established by implantation of NSCLC specimens of thirty-one patients into immunodeficient mice. Genetic aberrations were detected in six of the models, including one model with an *EGFR* activating mutation (Exon19 Del), one model with *KRAS* mutation, one model with both *KRAS* mutation and *cMET* gene amplification, and three models with *FGFR1* amplification. Anti-tumor efficacy studies using gefitinib demonstrated that the *EGFR* activating mutation model had superior sensitivity and that the *KRAS* mutation models were resistant to gefitinib. The range of gefitinib responses in the patient-derived NSCLC xenograft models were consistent with the results reported from clinical trials. Furthermore, we observed that patient-derived NSCLC models with *FGFR1* gene amplification were insensitive to gefitinib treatment.

**Conclusions:**

Ten patient-derived NSCLC xenograft models were established containing a variety of genetic aberrations including *EGFR* activating mutation, *KRAS* mutation, and *FGFR*1 and *cMET* amplification. Gefitinib anti-tumor efficacy in these patient-derived NSCLC xenografts containing *EGFR* and *KRAS* mutation was consistent with the reported results from previous clinical trials. Thus, data from our panel of patient-derived NSCLC xenograft models confirms the utility of these models in furthering our understanding of this disease and aiding the development of personalized therapies for NSCLC patients.

## Introduction

Lung cancer is the leading cause of cancer-related mortality worldwide [1] and non-small cell lung cancer (NSCLC) accounts for over 80% of lung cancer deaths [2]. In addition to the traditional lung cancer treatments of surgery, radiation and chemotherapy, molecularly targeted drugs such as *EGFR* tyrosine kinase inhibitors (gefitinib and erlotinib) [3], and anaplastic lymphoma kinase (ALK) tyrosine kinase inhibitors (crizotinib) [4] have recently emerged as viable therapeutic options.

Preclinical evaluation of targeted therapies relies heavily on the use of animal tumor models [5,6], and the transplantation of standard tumor cell lines into mice to generate xenografts is common practice in preclinical drug discovery [7,8]. Unfortunately, following prolonged *in vitro* artificial culturing, these transplanted tumor cells often no longer maintain the original molecular characteristics and heterogeneity of the patient tumor [9,10]. One of the most profound issues with using standard xenograft models is their poor predictive power for the translation of preclinical efficacy into clinical outcome [11,12].

To overcome these disadvantages and build upon the industry’s repertoire of standard xenograft tumor models, patient-derived xenograft mouse models have been successfully established by implanting fresh patient tumor fragments into immunodeficient mice, subcutaneously or orthotopically, and used to evaluate targeted therapeutic drugs in recent decades [5,12-14].

To date, a variety of tumor models representing key disease segments have been established using fresh patient tumor tissues, including colorectal cancer (CRC) [15], pancreatic cancer [16], lung cancer [17], gastric cancer [18], esophageal cancer [19], hepatocellular carcinoma (HCC) [20] and others [13]. Recent evidence suggests that patient-derived xenograft mouse models can maintain certain pathological and molecular features of the original disease and patient tumor [20]. Compared to standard cell line-derived xenograft models, the greatest advantage of patient-derived xenograft models is their ability to better predict clinical tumor response [6]. Notably, Fiebig *et al.* described a high correlation (90 ~ 96%) between anti-tumor efficacy generated in patient-derived xenograft models and clinical response in patients. In many respects, patient-derived tumor xenograft models are increasingly being considered as more relevant models since the patient’s tumor grows as a solid entity, develops a functional stroma and vasculature, and displays central necrosis and reflects tumor differentiation [6]. In a recent comprehensive review, the many advantages of patient-derived tumor xenograft models were summarized thus: 1) an accurate reflection of the complexity and heterogeneity of human tumors, 2) maintenance of the molecular, genetic and histological heterogeneity typical of the original tumors through serial passaging in mice, 3) provision of an excellent *in vivo* preclinical platform to study cancer stem biology and stromal-tumor interactions, 4) presentation of an information-rich preclinical resource for the analysis of drug activity, including novel-novel drug combinations, as well as predictive biomarker discovery, and finally 5) provision of a more-relevant system to test clinically directed hypotheses [21]. Thus, these clinically-relevant animal tumor models are anticipated to increase the success of identifying new active antitumor agents for targeted therapy. Furthermore, the increased genetic diversity within these patient-derived xenograft models also provides opportunities to further develop personalized approaches for the treatment of NSCLC patients.

To preclinically model the major disease segments within lung cancer, patient-derived NSCLC xenograft mouse models have been established, and further characterization has shown that these models accurately represent the patient’s genetic diversity and tumor morphology [17,22]. To our knowledge however, targeted therapeutic drugs such as the *EGFR* tyrosine kinase inhibitor gefitinib, have not been tested within a variety of patient-derived NSCLC xenograft models with differing genetic aberrations and compared with clinical trial outcome data.

In this study, we established ten passable patient-derived NSCLC xenograft models from thirty-one implanted patient NSCLC samples. These contained a variety of genetic aberrations including: *EGFR* and *KRAS* gene mutations and genetic amplification of *cMET* and *FGFR1*. Within these models, gefitinib antitumor activity was generally poor, and tumor regression was only observed in a model containing an *EGFR* exon 19 deletion - consistent with previous preclinical studies and supportive of the current patient selection criteria for *EGFR*-targeted therapies, such as gefitinib and erlotinib. These clinically relevant NSCLC xenograft models thus provide useful tools for the evaluation and development of molecularly targeted therapeutic drugs for the treatment of NSCLC patients.

## Materials and methods

### Patient samples

Thirty-one NSCLC patient specimens were obtained at initial surgery from primary diagnosed, early-stage NSCLC patients from Guangdong General Hospital. Written informed consent was obtained from each patient and the study was approved by the hospital ethics committee. Tumor pathology was diagnosed by hospital pathologist. Harvested fresh NSCLC specimens were separated into three parts: the first part was cut into fragments of around 15 mm^3^ under sterile conditions and put into frozen medium, containing 90% fetal bovine serum (FBS) (Gibco, Cat#10099-141, Australia) and 10% dimethyl sulfoxide (DMSO) (Sigma, Cat#D2650, USA), and then stored in liquid nitrogen for later implantation; the second part of the specimen was snap frozen immediately in liquid nitrogen for DNA/RNA extraction and the third part of the specimen was fixed in formalin (SCRC, Cat#10010018, China) and embedded into paraffin (Thermo Scientific, Cat#6774060, USA) for pathological assessment and model characterization.

### Establishment of patient-derived NSCLC xenograft models

8-10-week-old female severe combined immunodeficient (SCID) and nude (*nu/nu*) mice (Vital River, Beijing, China) were used in this study. All animal studies were performed in accordance with the guidelines approved by the Institutional Animal Care and Use Committee (IACUC). The patient-derived NSCLC xenograft mouse models were established using surgically removed tissues from NSCLC patients as described previously [5] and modified. Frozen NSCLC tissues in medium with 90% FBS were thawed at 37°C and implanted into SCID mice subcutaneously via Trocar needle. The tumor-implanted mice were observed daily for 90 days. Tumors were measured once a week by caliper to determine subcutaneous growth rate. Xenografted NSCLC tumors (~500 mm^3^) in SCID mice were further implanted into nude mice for further generations of these models. After three consecutive mouse-to-mouse passages, the xenograft models became stable and were then submitted for model characterization, including histopathology confirmation, gene mutation detection for *EGFR* and *KRAS*, and gene amplification for *FGFR1* and *cMET* by detection of fluorescence *in situ* hybridization (FISH) assay. The tumor specimens in each passage of tumor-bearing mice were harvested and divided into three parts for the following purposes. The first part was implanted into nude mice for generation of the second xenograft passage. The second part was used for DNA/RNA extraction by snap freezing in liquid nitrogen. The third part was fixed in 10% formalin buffer for 24 hours and embedded in paraffin (FFPE) for gene amplification by FISH analysis. Larger amounts of fresh tumor fragments at passage 3–5 were frozen in the standard cell freezing medium and stored in liquid nitrogen for model banking. The patient-derived NSCLC xenograft mouse models were maintained in nude mice and used for anti-tumor efficacy studies, prior to reaching passage ten. Hematoxylin and eosin (H&E) staining was performed to confirm the histopathology of xenografts.

### Mutation detection

NSCLC patient tissues and xenograft tissues from the patient-derived NSCLC xenograft mouse models were pathologically reviewed to ensure that tumor cell content was more than 80% and that no significant tumor necrosis had occurred before extraction of DNA. Genomic DNA was extracted from each tissue sample using Puregene Cell and Tissue Kit (QIAGEN, Cat#158388, Germany). The quantity and purity of DNA samples were measured using Nanodrop ND-1000 UV/VIS Spectrophotometer (Thermo Scientific, USA). DNA fragment integrity was confirmed by electrophoresis using 1% agarose gel. The concentration of DNA samples were normalized to 20 ng/μL and stored at −20°C until use. ‘Hot spot’ mutations in *EGFR* (exons 18, 19, 20, 21) and *KRAS* (exons 2 and 3) were screened by amplification refractory mutation system (ARMS) and mutant-enriched liquid chip polymerase chain reaction (PCR) method. The former method and detection was supported by Amoy diagnostics Co. Ltd, Fujian, PR China, the latter method was performed at SurExam Bio-Tech Co. Ltd., Guangzhou Technology Innovation Base, Science City, Guangzhou, PR China.

### *FGFR1* and *MET* gene amplification analysis by FISH assay

The *FGFR1* FISH probe was generated internally (AstraZeneca) by directly labeling bacteria artificial chromosome (BAC) (CTD-2288 L6, Invitrogen, USA) DNA with Spectrum Red (Vysis, Cat# 30–803400, USA). The CEP8- Spectrum Green probe (Vysis, Cat#32-132008, USA) for the centromeric region of chromosome (CEP) 8 was used as internal control. For *MET* FISH probe generation, BAC (CTD-2270 N20, Invitrogen, USA) DNA was used and CEP7-Spectrum Green probe (Vysis, Cat#06 J37-007, USA) as internal control.

FISH assays were performed on 4 micron dewaxed and dehydrated FFPE sections. The SpotLight Tissue pretreatment Kit (Invitrogen, Cat#00-8401, USA) was used for pretreatment (boiled in reagent 1 for ~15 minutes then coated with reagent 2 for ~10 minutes, minor time adjustments were made for individual samples). Sections and probes were codenaturated at 80°C for 5 minutes and then hybridized at 37°C for 48 hours. After a quick post wash off process (0.3%NP40/1xSSC at 75.5°C for 5 minutes, twice in 2 × saline sodium citrate [SSC] at room temperature for 2 minutes), sections were finally mounted with 0.3 μg/ml 4’,6-diamidino-2-phenylindole (DAPI) (Vector, Cat# H-1200, USA), and stored at 4°C avoiding light for at least 30 minutes prior to scoring.

Target gene and CEP signals were observed using fluorescence microscope equipped with the appropriate filters allowing visualization of the intense red target gene signals, the intense green chromosome centromere signals, and the blue counterstained nuclei. Enumeration of the *FGFR1* or *MET* gene and chromosome 8 or 7 was conducted by microscopic examination of 50 tumor nuclei, which yielded a ratio of *FGFR1* to CEP8 or *MET/*CEP7. Tumors with ratio ≥2 or presence of ≥10% gene cluster were defined as amplification.

### Anti-tumor activity

Tumor growth curves in all patient-derived NSCLC xenograft models were generated by kinetic measurement of tumor volumes subcutaneously. For therapeutic experiments, the tumor volume range of 150 to 250 mm^3^ in tumor-bearing nude mice were sorted randomly (6 ~ 8 animals per group) and assigned to vehicle control or gefitinib treatment groups by oral dosing at 100 mg/kg (AstraZeneca, AZ10027436, England). Subcutaneous tumor volumes in nude mice and mouse body weights were measured twice a week. Tumor volumes were calculated by measuring two perpendicular diameters with calipers. Tumor volumes (TV) calculated by the formula: TV = (length × width^2^)/2. Percentage of tumor growth inhibition (%TGI) was calculated as the formula: {1 – [change of tumor volume in treatment group/change of tumor volume in control group]} × 100 and was used for the evaluation of anti-tumor efficacy.

### Statistical analysis

To find out clinical parameters that contribute to the success of model establishment, logistic regression was used to assess the association of success rate of model establishment with clinic-pathological parameters. A patient tissue that can be successfully turned into a xenograft model is defined as 1 and 0 otherwise. P values from univariate models were computed from log-likelihood ratio test. Factors that show significant results from univariate analysis were considered in multivariate analysis to adjust for imbalance of covariates, including gender, histological type and smoking status. The data analysis was performed using R version 2.11.0 on Unix. To evaluate the statistic significance in anti-tumor efficacy study, Student’s *t-test* was used to compare TGI in treatment group to the control group. Statistical tests were two sided, with P < 0.05 considered significant.

## Results

Establishment of patient-derived NSCLC xenograft mouse models.

**Figure 1:**
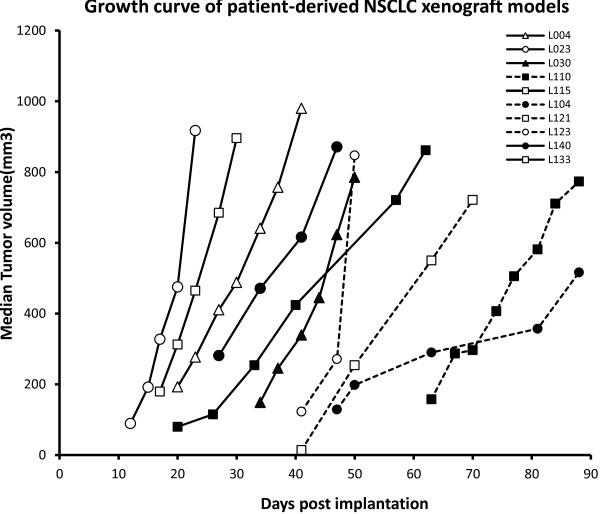
***In vivo *growth curves of patient-derived NSCLC xenograft models.** Tumor growth curves of patient-derived NSCLC xenograft models. The models were established subcutaneously and tumor growth curves in passable and stable xenograft models were generated by tumor measurement between passage 3–5 (F3-5). Media of tumor volume was showed.

Thirty-one patient NSCLC specimens were harvested and implanted subcutaneously into SCID mice. Ten patient-derived NSCLC xenograft models from the thirty-one implantations were established in consecutive passages in nude mice (Table 1). NSCLC patient clinical information is also listed in Table 1. None of the patients received any therapies prior to surgery. The original patient NSCLC tissues were implanted into SCID mice subcutaneously and then growing xenograft tissues were implanted into nude mice starting from the second generation of models. The model success rate was 45% (14/31) in the first generation of SCID mice, and then 32% (10/31) in the second and subsequent generations. After the second generation, patient-derived NSCLC xenograft models became stable without further changes in model survival and tumor take-on rates. All ten established patient-derived NSCLC xenograft models showed kinetic growth curves in passage 3 (Figure 1). Examination of autopsies in NSCLC-bearing mice from these xenograft mouse models at 2 ~ 3 months post-implantation revealed no evidence of metastases in brain, lung, liver or kidney. The xenograft tissues were analyzed by H&E staining for pathology assessment. The patient-derived NSCLC xenograft tissues exhibited similar morphology to that of the patient tissues from which the primary models were derived (data not shown). Following this, established patient NSCLC xenografts were submitted for model characterization and further validation by targeted therapy.

**Table 1:** Information of patients and corresponding patient-derived xenograft mouse models

**#**	**Model**	**Gender**	**Smoking status**	***EGFR/Kras *mutation**	**Age**	**Pathology**	**Stage**	**TNM stage**	**Engrafted model**
**1**	L004	M	Y	WT	61	AC	IIIA	T2N2M0	Yes
**2**	L023	M	Y	Kras:G12S	55	SCC	IIA	T3N2M0	Yes
**3**	L030	M	Y	Q61H	64	SCC	IV	T2N0M1	Yes
**4**	L101	M	Y	WT	63	AC	IA	T1N0M0	No
**5**	L102	F	N	WT	53	AC	IA	T1N0M0	No
**6**	L103	M	N	n/a	45	SCC	IIA	T3N0M0	No
**7**	L104	M	N	WT	71	SCC	IIB	T2N1M0	Yes
**8**	L105	M	N	WT	76	AC	IB	T2N0M0	No
**9**	L106	F	N	Exon19Del	67	AC	IIB	T2N1M0	No
**10**	L107	M	Y	WT	76	SCC	IB	T2N0M0	No
**11**	L108	F	N	L858R	71	AC	IIB	T2N1M0	No
**12**	L110	M	Y	WT	66	SCC	IB	T2N0M0	Yes
**13**	L111	M	N	L858R	72	AC	IA	T1N0M0	No
**14**	L113	M	Y	WT	73	SCC	IB	CT2N0M0	No
**15**	L115	M	n/a	Exon19Del	55	AC/SCC	IIIA	T2N2M0	Yes
**16**	L116	M	N	WT	57	SCC	IIIA	ST2N2M0	No
**17**	L117	M	N	WT	66	AC	IIB	ST2N1M0	No
**18**	L118	M	Y	L858R	31	AC	IV	T1N0M1	No
**19**	L119	M	Y	WT	48	AC	IA	ST1N0M0	No
**20**	L121	M	Y	WT	69	SCC	IV	T2N0M1	Yes
**21**	L123	M	Y	WT	50	SCC	IB	T2N0M0	Yes
**22**	L124	F	N	L858R	50	AC	IIIA	T2N2Mx	No
**23**	L125	F	N	WT	47	AC	IIIA	T2N2M0	No
**24**	L126	M	N	WT	59	AC	IB	T2N0M0	No
**25**	L127	F	N	L858R	66	AC	IB	T2N0M0	No
**26**	L128	M	Y	WT	61	SCC	IIIA	T2N2M0	No
**27**	L130	M	N	n/a	66	SCC	IB	T2N0M0	No
**28**	L131	F	N	Kras:G12V	60	AC	IV	N0M1	No
**29**	L132	M	Y	WT	69	AC	IIIA	ST3N1M0	No
**30**	L133	M	N	WT	61	SCC	IIB	T2N1M0	Yes
**31**	L140	M	Y	WT	58	SCC	IIIA	T2N2M0	Yes

Results of the correlation analysis between model establishment success rate and patient clinical parameters are listed in Table 2. Multivariate analysis showed that tumor histological subtype was the only parameter which had significant impact on the success rate of model establishment. Squamous cell carcinoma (SCC) was much more prone to be tumorigenic in nude mice compared to adenocarcinoma (AC). Other factors, including sex, smoking status, pathologic grade and mutation, did not correlate with model success rate; although univariate analysis showed that sex and smoking status have a significant impact when these parameters are considered alone.

**Table 2:** Relationship of model establishment rate and patients’ clinical information

**Patient information**	**Patient #**	**Derived model #**	**Established rate (%)**	***P *value (univariate)**	***P *value (multivariate)**
**Sex**				0.0115	0.1896
Male	24	10	42		
Female	7	0	0		
**Histologic type**				0.0015	0.0218
SCC	15	9	60		
AC	16	1	6		
**Pathologic stage**				0.5017	n/a
Stage I	12	2	17		
Stage II	7	3	43		
Stage III/IV	11	4	36		
**Smoke status**				0.0228	0.4631
Smoker	14	7	50		
Non-smoker	16	2	12.5		
**Mutation**				0.1031	n/a
*EGFR*	7	1	14		
*KRAS*	3	2	67		

### Genetic aberrations within patient-derived NSCLC xenograft models

**Figure 2:**
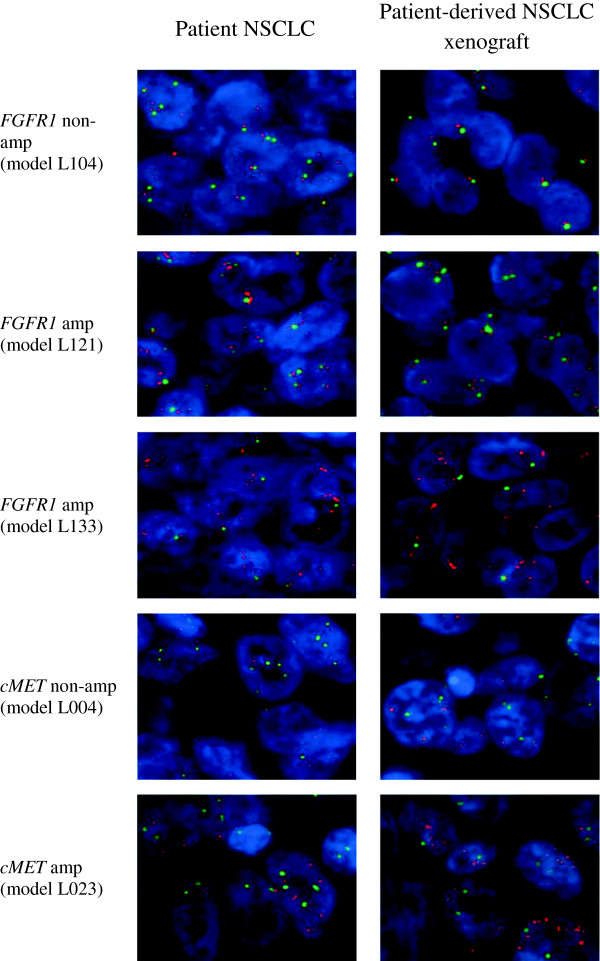
**Representative pictures of FISH imaging on patient-derived NSCLC xenograft models L004, L023, L104, L121, L133 with matched human primary tumor tissues.***FGFR* and *cMET* gene amplifications were detected in models L121/L133, and L023, respectively. Non-*FGFR* gene and non-*cMET* gene amplification were detected in models L104 and L004, respectively.

Mutation of *EGFR, KRAS* and gene amplification of *FGFR1* and *cMET* were screened for in all 31 patient samples and ten established xenograft samples. One model with an *EGFR* activating mutation (Exon19Del) and two models with *KRAS* mutations (G12S and Q61H) were identified in the ten patient-derived NSCLC models and their corresponding patient NSCLC tissues (Table 3). Furthermore, gene amplification of *FGFR1* was detected in additional three models and *cMET* in one model (*KRAS* mutant background). Again, these genetic aberrations were identified in both patient-derived NSCLC models and their corresponding patient NSCLC tissues (Table 3 and Figure 2), with the exception of one model (L123) which lacked sufficient patient LC tissue for analysis.

**Table 3:** Patient samples and corresponding patient-derived NSCLC xenograft model information

**Model ID**	**Patient NSCLC tissue implanted**					**Patient derived LC xenograft models**				
	**Pathology**	**Mutation detection**		**Gene amplification**		**Mutation detection**		**Gene amplification**		**% TGI**
		***EGFR***	***KRAS***	***FGFR1***	***MET***	***EGFR***	***KRAS***	***FGFR1***	***MET***	**by Gefitinib**
**L004**	AC	WT	WT	None	None	WT	WT	None	None	113
**L023**	SCC	WT	G12S	None	AMP	WT	G12S	None	AMP	0
**L030**	SCC	WT	Q61H	None	None	WT	Q61H	None	None	25
**L104**	SCC	WT	WT	None	None	WT	WT	None	None	46
**L110**	SCC	WT	WT	None	None	WT	WT	None	None	78
**L115**	SCC	Exon19Del	WT	None	None	Exon19Del	WT	None	None	199
**L121**	SCC	WT	WT	AMP	None	WT	WT	AMP	None	23
**L123**	SCC	WT	WT	N/A	None	WT	WT	AMP	None	47
**L133**	SCC	WT	WT	AMP	None	WT	WT	AMP	None	N/A
**L140**	SCC	WT	WT	None	None	WT	WT	None	None	N/A

### Gefitinib antitumor activity

**Figure 3:**
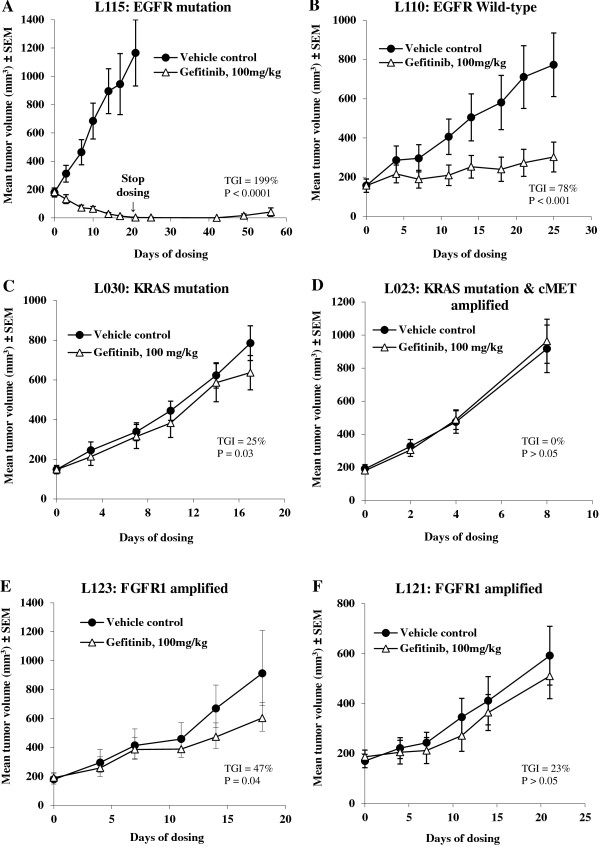
**Efficacy study in patient-derived NSCLC xenograft models.** Tumor-bearing nude mice were treated with either vehicle control or gefitinib at 100 mg/kg orally daily dosing for 1 ~ 3 weeks based on tumor growth rates in different patient-derived NSCLC xenograft models when tumors reached 150 ~ 250 mm^3^ post implantation. Genetic aberrations within each of the models **(A-F)** are labeled in the graph title. Within the models, *EGFR*, *KRAS*, *MET* and *FGFR1* are wild-type and non-amplified unless otherwise stated in the graph title.

To validate and confirm whether these patient-derived NSCLC xenograft models showed similar responses to gefitinib treatment as compared to that reported in clinical trials, eight of the ten established xenograft models were treated with the *EGFR* tyrosine kinase inhibitor gefitinib (Table 3 and Figure 3). Xenograft model L115 which harbored an *EGFR* activating mutation (Exon19Del), responded completely to gefitinib treatment (tumors regressed completely during the treatment period). Interestingly, these tumors remained almost indistinguishable for nearly 40 days following cessation of gefitinib dosing. Xenograft model L030, with a *KRAS* mutation and *EGFR* wild type gene, showed a minor response to gefitinib treatment (25% tumor growth inhibition (TGI)). A further *KRAS* mutation model (L023) with *cMET* gene amplification had no response to gefitinib treatment. Not surprisingly, the patient-derived NSCLC xenograft models, L121 & L123 with *FGFR1* gene amplification were insensitive to gefitinib treatment (Table 3 and Figure 3), likely due to a tumor-dependence on *FGFR* pathway signaling [23,24]. Additional models, L104 and L004 with wild type *EGFR* and *KRAS* but without *FGFR1* and *cMET* gene amplification showed moderate response to gefitinib, ranging from 46% to 113% TGI (Table 3 and Figure 3).

## Discussion

As NSCLC remains one of the most lethal malignancies worldwide [1,2,25], we urgently need to improve our understanding of the disease and assess whether novel patient-derived NSCLC xenograft models can more faithfully represent clinical disease and gauge response to targeted therapies. We established ten patient-derived NSCLC xenograft mouse models derived from frozen patient tissues and characterized these models for relevant gene mutations and amplifications. In previous reports, patient-derived NSCLC xenograft models were established by implanting fresh patient tumor tissues with success rates ranging from 25 ~ 40% [5,22]. To establish whether frozen tumor tissues could also be used to generate patient-derived xenograft models, we directly implanted frozen patient NSCLC tissues into mice. Using this method, we achieved a success rate of 32% (10/31 implantation), within a similar range to that achieved using fresh tissue implantation. An obvious benefit of this method however, is that patient tumor tissues can be collected at multiple medical centers, frozen on site and shipped to a central animal facility for model establishment, characterization and testing.

Here, as with others [5,13], we have shown that implantation of human NSCLC tissue (in our case frozen tissue) can be viable and lead to successful model development in SCID mice. In our experience however, nude mice models are preferable in drug discovery due to their superior tolerability of drug agents and hence, we transferred our established patient-derived NSCLC models from SCID to nude mice. Our studies demonstrated that 71% (10/14) of our xenograft models in SCID mice could be successfully transferred to nude mice starting from the 2nd generation. After the third generation, in our study the models became more stable in terms of both tumor take-on and success rates.

To date, the major factors which affect the success rates of establishing patient-derived xenograft models in mice are still unclear. We suggest that tumor type may be one of the key factors. Clearly, SCC is easier to grow up in mice than AC [22], and higher success rates have been observed in establishing SCC patient-derived xenograft tumor models compared to all other types of patient-derived tumor [22]. Thomas John *et al.* have recently demonstrated that patient samples with *EGFR* gene mutations are difficult to grow up and passage in immunodeficient mice. Our data in this study also support this observation (Table 2). Clinical characteristics, such as patient clinical stage, pathological grade and gender showed no correlation with the success rates of the patient-derived NSCLC xenograft models. This is a similar observation to those of other investigators in this field [5,14,26,27]. However, patient tumors with late stage and higher tumor grade tended to be passaged further in mice [26]. On the other hand, univariate analysis showed that sex and smoking status both significantly affected the success rate of patient-derived xenograft model establishment, however multivariate analysis failed to show this correlation. This result implies that males and smokers are more prone to developing SCC, and consequently that female patients and non-smokers are more prone to developing AC. Furthermore, we observed that the quality of the tumor samples provided by surgeons was one of the most important factors for successful engraftment of patient-derived tumor xenograft models (unpublished ongoing studies).

One of the most important advantages in developing patient-derived NSCLC xenograft models is that the model can better represent the genetic diversity and molecular characteristics of the original NSCLC patient tumor [22]. With regard to the carryover of genetic mutations from patient NSCLC tissues to mouse xenograft tissues, data from our study showed that genotypes are consistent between the original patient tumor and the corresponding xenograft tissue in all ten models for *EGFR* and *KRAS* status. In addition to mutations, our data also demonstrated that these patient-derived NSCLC xenograft models carry gene amplifications in 4/10 models, including *FGFR1* and *cMET*, which are known anti-tumor drug targets [28-30]. Thus, these novel xenograft models represent useful tools for the preclinical study of emerging targeted therapies.

Although the work of Judde *et al.* demonstrated a gefitinib anti-tumor response in a patient-derived NSCLC model (58% TGI) [31], genetic aberrations were not explored and characterized in detail across multiple models. In an effort to verify whether these novel models with different genetic aberrations exhibited a similar anti-tumor response to targeted therapy, we tested the efficacy of gefitinib in model L115, harboring an exon 19 deletion within *EGFR*. Gefitinib is an anti-*EGFR* agent shown to have clinical activity for the treatment of *EGFR* mutation positive NSCLC patients [32]. In clinical studies, gefitinib induces initial tumor regressions in lung cancer patients with *EGFR* activating mutations, including Exon19 Del, L858R mutation, and has no significant anti-tumor activity in lung cancer patients with *KRAS* mutation [32]. Our data confirmed a very consistent response profile in the patient-derived NSCLC models. Model L115 responded completely to gefitinib treatment over 20 days of dosing (tumor disappeared), and displayed only very slow regrowth after 38 days post-dose cessation (Figure 3A). Two of the models with *KRAS* mutation were insensitive to gefitinib treatment. Interestingly, one *KRAS* mutation model, L030 showed little response to gefitinib (25% TGI), but the other, L1023 (*KRAS* mutation concurrent with *cMET* gene amplification) showed no response at all (0% TGI in L023 model).

*FGFR1* represents a promising new target in lung cancer therapy [28]. Here, we tested two of three *FGFR1* gene amplified models derived from NSCLC patients with gefitinib. Compared to the *EGFR* activating mutation model, these two *FGFR1* gene amplified models, L121 and L123 were relatively insensitive to gefitinib treatment (TGI = 23%, and 47%, respectively). These models are likely ‘oncogene addicted’ to *FGFR1* and indeed, potent tumor regressions have been previously observed in these 2 models using the selective small molecule inhibitor, AZD4547 [24]. A third *FGFR1* amplified model, L133, failed to show a potent response to AZD4547 due to a lack of FGFR1 protein expression. Without question, these patient-derived NSCLC xenograft models harboring *FGFR1* gene amplification have been extremely useful tools in testing the hypothesis that *FGFR1* is a driving oncogene in NSCLC.

In summary, ten patient-derived NSCLC xenograft models were established harboring a variety of genetic aberrations including; *EGFR* activating and *KRAS* mutations, and *FGFR1* and *cMET* gene amplification. Within those models with *EGFR* and *KRAS* mutation, the anti-tumor efficacy of the *EGFR* tyrosine kinase inhibitor, gefitinib, was consistent with published preclinical data [33] and clinical responses [34-36]. Finally, these patient-derived NSCLC xenograft models represent useful tools to further understand this lethal disease and to enable development of personalized approaches for the treatment of NSCLC patients.

## Competing interests

The authors declare that they have no competing interests.

## Authors’ contributions

XZ and JZ participated in study design and coordination and drafted the manuscript. XH, ZY and WZ collected and characterized the fresh patient tumor tissues. ML, LX, LZ and MZ performed the establishment of animal tumor models and efficacy study. GZ participated in the sequence alignment. LZ and XS completed immunochemistry and the FISH studies. PZ performed the statistical analysis. PG revised the manuscript. YW and QJ conceived of the study and participated in its design. All authors read and approved the final manuscript.
